# Three-Dimensional Postmortem Ultrasound of the Fetal Corneal Volume to Estimate Postmortem Interval

**DOI:** 10.3390/jcm15051865

**Published:** 2026-02-28

**Authors:** Patricia Ibarra Vilar, Dominique A. Badr, Laura De Luca, Teresa Cos Sanchez, Jacques C. Jani, Xin Kang

**Affiliations:** Department of Obstetrics and Gynecology, University Hospital Brugmann, Université Libre de Bruxelles, Place A. Van Gehuchten 4, 1020 Brussels, Belgium; patricia.ibarravilar@chu-brugmann.be (P.I.V.); dominique.badr@chu-brugmann.be (D.A.B.); laura.deluca@chu-brugmann.be (L.D.L.); teresa.cossanchez@chu-brugmann.be (T.C.S.); jacques.jani@chu-brugmann.be (J.C.J.)

**Keywords:** postmortem interval, fetal death, three-dimensional ultrasound, postmortem imaging, cornea, machine learning

## Abstract

**Objectives**: To develop and prospectively validate a predictive model to estimate the fetal postmortem interval (PMI) using three-dimensional postmortem ultrasound (3D PM-US) measurements of corneal and ocular volumes. **Methods**: Single-center study including fetuses ≥ 20 weeks’ gestation with known time of death after feticide. A retrospective training cohort (*n* = 63; November 2022–July 2023) and a prospective validation cohort (*n* = 28; February–August 2025) were used. Corneal and ocular volumes were measured using the VOCAL™ rotation multiplanar technique; the cornea-to-eyeball volume ratio was calculated for each case. Automated machine learning (AutoML) was used to develop and validate a gradient boosting machine (GBM) model. Model performance was evaluated using the root mean squared error (RMSE), mean absolute error (MAE), and coefficient of determination (R^2^). **Results**: Ninety-four fetuses were included; three were excluded (two for extreme death–US intervals of 165 and 166 h; one for open eyelids). Median gestational age was 29.3 weeks (IQR 27.2–32.9); median birthweight was 1325 g (IQR 980–1880). The cornea-to-eyeball volume ratio was an independent predictor of PMI (*p* < 0.001). The GBM model explained 91% of the variance in the training cohort (R^2^ = 0.91, RMSE = 11.49 h, MAE = 8.45 h) and 75% in the validation cohort (R^2^ = 0.75, RMSE = 18.32 h, MAE = 14.49 h), demonstrating strong predictive accuracy and minimal overfitting. Variable importance analysis confirmed the cornea-to-eyeball ratio as the most influential and biologically plausible predictor of PMI. A Shiny web application was developed to facilitate clinical implementation. **Conclusions**: 3D PM-US measurements of the fetal cornea and eyeball can reliably and quantitatively estimate the PMI with good predictive accuracy using a GBM model. Multicenter studies are required to further refine the model, enable external validation, and determine its clinical and forensic utility.

## 1. Introduction

Stillbirth remains one of the most distressing outcomes in obstetrics, affecting nearly two million families worldwide each year, with approximately 1.9 million stillbirths reported globally in 2021 [1]. Accurate estimation of the fetal postmortem interval (PMI) is crucial to understand the circumstances of death, establish the cause of the fetal demise, and counsel parents regarding future pregnancies. Current approaches—including maternal recall of fetal movements, last documented fetal heart activity, and maceration scoring—are subjective and imprecise [2], with poor reproducibility across gestational ages and intrauterine conditions.

Postmortem changes in fetuses have been studied using macroscopic [3,4] and histologic [5,6,7] markers to estimate PMI, yet none have achieved consistent accuracy. Maceration scoring, for instance, reflects intrauterine retention and tissue autolysis but is influenced by gestational age, retention time, and intrauterine conditions [8]. Conventional autopsy, long considered the gold standard, has increasingly declined due to parental preference or cultural and religious reasons, limiting access to definitive postmortem findings [9,10]. Non-invasive virtual autopsy using postmortem magnetic resonance imaging (MRI) has also been explored, focusing on quantitative parameters such as apparent diffusion coefficient (ADC) values of fetal organs [11,12,13,14,15]. However, these studies have shown high interindividual variability and inconsistent correlations with PMI, limiting their clinical utility. To date, no invasive or imaging-based approach has achieved reproducible accuracy for fetal PMI estimation in human subjects.

In adult forensic research, ocular structures, particularly the cornea, have been identified as potential indicators of PMI. Corneal opacity develops progressively after death due to endothelial degeneration and aqueous humor diffusion, and its correlation with PMI has been demonstrated through macroscopic and microscopic evaluation [16], cytological assessment of postmortem epithelial degeneration [17], and quantitative image analysis in adults human subjects [18]. Therefore, three-dimensional postmortem ultrasound (3D PM-US) of the fetal cornea and eyeball may provide a quantitative, non-invasive tool for estimating fetal PMI.

The objective of this study was to evaluate the association between fetal corneal volume and PMI, and to develop a machine learning model to estimate PMI in fetuses > 20 weeks of gestation.

## 2. Materials and Methods

### 2.1. Study Design and Setting

This single-center study included a retrospective cohort for model development (November 2022–July 2023) and a prospective cohort for validation (February–August 2025). Both cohorts were recruited at the Fetal Medicine Unit, University Hospital Brugmann, Université Libre de Bruxelles, Brussels, Belgium—a tertiary referral center for fetal anomalies. The study was conducted according to the guidelines of the Declaration of Helsinki and approved by the institutional ethics committee (University Hospital Brugmann, CE: 2012/116, date of approval 30 October 2012, and CE:2021/108, date of approval 13 July 2021). Written informed consent was obtained from all parents for research use of anonymized postmortem imaging and, when accepted, invasive autopsy. The study followed the Transparent Reporting of a multivariable prediction model for Individual Prognosis Or Diagnosis (TRIPOD) guidelines [19].

### 2.2. Participants

Following termination of pregnancy (TOP), intrauterine fetal death (IUFD), or miscarriage, parents were counseled and offered conventional autopsy and/or virtual autopsy. Postmortem ultrasound (PM-US) has been a standard component of the virtual autopsy protocol at our institution since 2014 [20,21]. After delivery, all fetuses were stored at 4 °C until imaging.

Inclusion criteria were TOP at ≥20 weeks’ gestation with confirmed feticide to ensure accurate determination of the death-to-imaging interval. Exclusion criteria included TOP < 20 weeks without feticide, spontaneous stillbirths, miscarriages, incomplete datasets, or suboptimal ocular imaging quality.

### 2.3. Termination of Pregnancy

Feticide procedures were performed by senior fetal medicine specialists under ultrasound guidance. The umbilical vein at the placental insertion site was punctured, and 2% lidocaine (7–30 mL, adjusted for GA) was injected until cessation of fetal cardiac activity. If the umbilical vein was inaccessible, intracardiac injection was performed as an alternative. Date and time of the feticide were recorded.

### 2.4. Postmortem Ultrasound

#### 2.4.1. Equipment

PM-US examinations were performed by fetal medicine specialists with over 6 years of prenatal imaging experience and at least 3 years of PM-US experience. Imaging was performed using either a *SAMSUNG WS80 system* (Version 4, Seoul, Republic of Korea) equipped with a high-frequency linear probe LV3-14A (3–14 MHz; field of view: 38.4 mm) and a vaginal probe V5-9 (5–9 MHz; field of view 150.6°, volume angle 90°) or a *VOLUSON E8 system* (GE Medical Systems, Zipf, Austria) with a high-frequency linear probe RSP 6-16 (6–18 MHz; field of view, 37.4 mm; volume, 37.4 × 29°).

#### 2.4.2. Procedure

**Figure 1:**
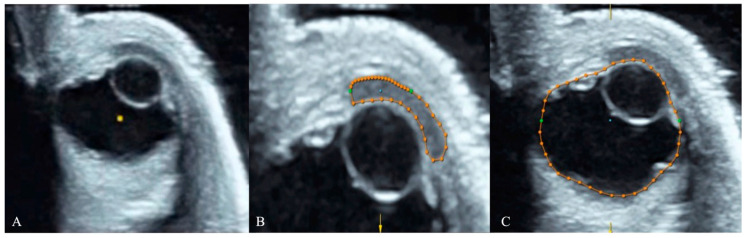
Three-dimensional postmortem ultrasound (3D PM-US) of the fetal orbit (**A**) showing multiplanar VOCAL™ analysis for quantitative volumetry of the cornea (**B**) and the eyeball (**C**). The contours were manually traced in six rotational planes spaced 30° apart to compute corneal and ocular volumes for ratio calculation.

Each fetus was placed supine in a small water bath, completely covered by a 2 cm water layer, and scanned with the probe partially immersed [22]. The transducer was positioned perpendicular to the fetal eyes to obtain an axial view of one orbit. The operator maintained a steady hand to prevent motion artifacts. The 3D volume included both orbits and surrounding structures. During post-processing, rendering was removed to allow detailed multiplanar analysis, and the axial plane was adjusted until a complete and well-defined image of the cornea, lens, and globe was visualized (Figure 1).

### 2.5. Ocular Volume Measurement

**Figure 2:**
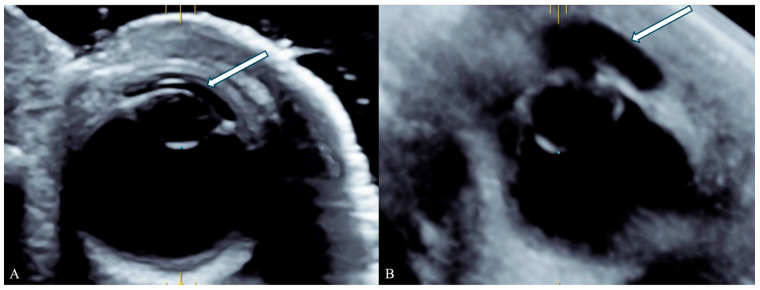
Comparison of three-dimensional postmortem ultrasound (3D PM-US) images of the fetal eye. The cornea (arrow) is located anterior to the lens, with the globe situated posteriorly. (**A**) Fetus examined 7 h after death shows a thin, well-defined cornea and a round, intact globe. (**B**) Fetus examined 144 h after death shows corneal thickening and irregularity, with partial collapse of the globe, illustrating the progressive postmortem changes in ocular morphology associated with increasing postmortem interval.

Ocular and corneal volumes were measured using the VOCAL™ rotational multiplanar technique (*Virtual Organ Computer-aided Analysis*) with system-specific software. For each fetus, the examiner selected the eye with the best image quality and measured the cornea and its corresponding eyeball on the same side. A single examiner (P.I.), blinded to clinical and postmortem information, manually traced the contours of both structures in six rotational planes spaced 30° apart. The reference plane included the largest anteroposterior diameters in the axial view. Volumes were automatically computed, and the cornea-to-eyeball volume ratio was calculated for each case (Figure 1 and Figure 2). For each fetus, the volume was measured twice and the mean of the 2 measurments were used for analysis. This approach minimizes inter-eye variability and ensures measurement consistency.

### 2.6. Statistical Analysis

All analyses were performed using R software (version 4.4.2; R Foundation for Statistical Computing, Vienna, Austria). Continuous variables were summarized as medians (interquartile ranges [IQR]) and categorical variables as counts and percentages.

To improve normality, a square-root transformation was applied to the delivery–US interval. Continuous predictors were standardized (z-scores) before analysis. Correlations between predictors were assessed using Pearson correlation coefficients.

The primary outcome variable was the *interval between fetal death and PM-US (death–US interval)*. Predictor variables included gestational age (GA), birthweight (BW), fetal gender, the cornea-to-eyeball volume ratio, and the delivery–US interval.

The sample size was determined by the number of consecutive eligible patients available during the study period. As this was a prediction modeling study rather than a hypothesis-testing study, no formal power calculation was performed. In the derivation cohort (*n* = 63), five predictors were included, corresponding to approximately 12 observations per predictor, which is consistent with commonly cited recommendations for multivariable model development.

Automated machine learning (AutoML; H2O.ai, Mountain View, CA, USA) was used to develop regression models predicting the death–US interval. Data were converted to H2OFrame objects, with the training frame comprising the training cohort and the testing frame comprising the validation cohort. AutoML trained up to 20 base models using 5-fold cross-validation within 120 s, exploring multiple algorithms and hyperparameters to select the best model on the external test (leaderboard) data. AutoML returned a leaderboard ranked by test performance and a leader model. We reported the leader’s test performance (on validation cohort) and summarized its internal cross-validation performance. Model performance was evaluated using root mean squared error (RMSE), mean absolute error (MAE), coefficient of determination (R^2^), mean square error (MSE), and root mean squared logarithmic error (RMSLE). Calibration was assessed with observed versus predicted plots.

To facilitate model deployment and individualized prediction, a custom Shiny application was developed in R. This application allows users to: upload new patient data (either raw or standardized values), generate model-based predictions using the best-performing H2O Gradient Boosting Machine (GBM) algorithm, visualize model explainability through Shapley Additive Explanations (SHAP), and display global feature importance and partial dependence plots interactively.

## 3. Results

A total of 94 fetuses were included: 63 in the training cohort and 31 in the validation cohort. Three fetuses were excluded: two due to excessively long death–US intervals (165 and 166 h), which resulted in extreme cornea-to-eyeball ratios, and one because open eyelids prevented adequate corneal imaging. Data from 28 fetuses were therefore available for model testing.

The median gestational age at delivery was 29.3 weeks (IQR, 27.2–32.9), and the median birthweight was 1325 g (IQR, 980–1880). Male fetuses represented 47.3% (43/91). The median death–US interval was 71.2 h (IQR, 51.0–115.1; range, 7.5–146.9 h). Median eyeball and corneal volumes were 0.80 cm^3^ (IQR, 0.59–0.97) and 0.06 cm^3^ (IQR, 0.04–0.08), respectively, yielding a median cornea-to-eyeball ratio of 0.07 (IQR, 0.06–0.10). Baseline distributions of gestational age, birthweight, and ocular measurements were comparable between cohorts (Table 1 and Table 2).

**Figure 3:**
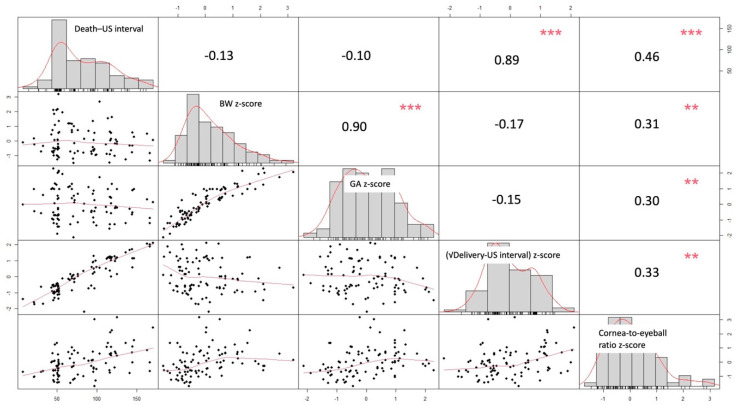
Pairwise correlation matrix among key study variables. Diagonal panels display the distribution (histogram and kernel density estimate) of each variable: interval between fetal death and ultrasound (death–US interval), birthweight z-score (BW z-score), gestational age z-score (GA z-score), square-root-transformed interval between delivery and ultrasound ((√delivery–US interval) z-score), and cornea-to-eyeball ratio z-score. Lower panels show scatterplots with locally weighted regression (LOESS) smoothers illustrating the relationships between variables. Upper panels present Pearson correlation coefficients, with the magnitude of association indicated by the font size. Scheme 0. ** *p* < 0.01, *** *p* < 0.001.

Correlation analysis among continuous variables showed strong association between gestational age and birthweight (r = 0.90, *p* < 0.001) and between the death–US and delivery–US intervals (r = 0.89, *p* < 0.001). The cornea-to-eyeball ratio demonstrated weak correlations with other variables (|r|< 0.46), supporting its independence as a predictor (Figure 3).

**Figure 4:**
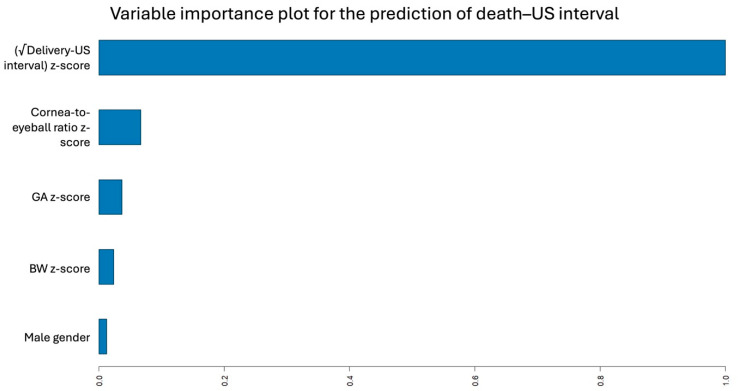
Variable importance plot for the prediction of the interval between fetal death and ultrasound examination (death–US interval). Variable importance reflects each predictor’s relative contribution to the model’s overall predictive performance, normalized to a maximum value of 1.0.

Automated machine learning (AutoML) identified a GBM as the best-performing regression model for predicting the death–US interval. The final model consisted of 44 trees with a maximum depth of 4 (mean depth, 2.68) and a mean of 4.1 terminal leaves per tree. Global variable importance ranked the delivery–US interval as the most modifiable predictor for death–US interval. The first non-modifiable predictor was cornea-to-eyeball ratio, followed by gestational age and birthweight, then male gender (Figure 4).

In the training dataset (*n* = 63), model performance metrics were: MSE = 132.1 h, RMSE = 11.49 h, MAE = 8.45 h, R^2^ = 0.91, and RMSLE = 0.164.

On 5-fold cross-validation data, the model achieved an MSE of 324.3 h, RMSE of 18.01 h, MAE of 13.73 h, and RMSLE of 0.238.

**Figure 5:**
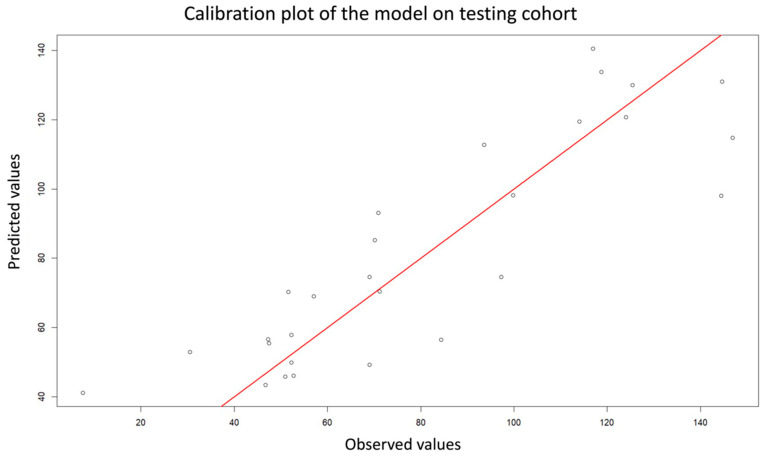
Calibration plot of the model in the testing cohort. The plot compares predicted versus observed values for the death–US interval to assess model calibration. Each point represents one observation from the testing dataset. The red line indicates the line of identity (perfect calibration), where predicted and observed values are equal.

**Figure 6:**
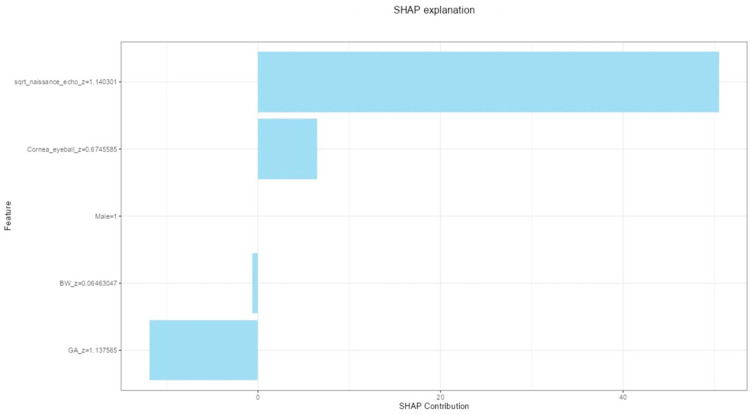
SHAP (SHapley Additive exPlanations) for an individual case with a male fetus, delivery-US interval of 91.033 h, cornea-to-eyeball volume ratio of 0.092, GA at delivery of 33.286, and BW of 1450 g. Each bar represents the contribution of a specific variable to the predicted value of the *death–US interval*. Positive SHAP values indicate features that increased the model’s prediction, whereas negative values indicate features that decreased it. The model estimated a *death–US interval* of 126.865 h, whereas the observed interval was 125.450 h.

In the validation cohort (*n* = 28), the model showed a modest decrease in accuracy but maintained good generalization and minimal overfitting (R^2^ = 0.75, RMSE = 18.32 h, MAE = 14.49 h). Calibration and dispersion were illustrated using observed-versus-predicted plots (Figure 5 and Figure 6).

## 4. Discussion

### 4.1. Principal Findings

In this study, we developed and prospectively validated a novel predictive model for estimating the fetal death–US interval using 3D PM-US measurements of corneal and ocular volumes in fetuses > 20 weeks’ gestation. The GBM model demonstrated good performance in the retrospective training cohort and maintained strong predictive accuracy in the prospective validation cohort. A mean absolute error of approximately 14 h represents a clinically acceptable margin, supporting the feasibility of 3D PM-US as an objective, reproducible, and non-invasive method for estimating the PMI.

### 4.2. Interpretation in the Context of Existing Evidence

To our knowledge, this is the first study to demonstrate that quantitative PM-US data can accurately predict PMI in human fetuses. Prior investigations using PM-MRI have explored diffusion-related biomarkers, such as apparent diffusion coefficient (ADC) values of fetal organs including the lungs and brain [11,12,13,14,15]. Although early data suggested a negative correlation between ADC and PMI, subsequent studies showed inconsistent results, likely due to tissue heterogeneity, environmental variability and methodological limitations. PM-MRI also requires expensive equipment and advanced technical expertise, limiting its availability. Our approach provides a methodological advance by combining widely accessible imaging technology with interpretable machine learning techniques to obtain objective and reproducible PMI estimates.

3D PM-US has multiple advantages: it is non-invasive, inexpensive, reproducible, and widely available. Image acquisition is rapid, and volumetric quantification requires only basic operator training, with a short learning curve. Because it does not require specialized equipment or prolonged examination times [22], this method is suitable for both high-resource and low-resource settings and for parents declining conventional autopsy. Importantly, the reproducibility of corneal and ocular measurements across different ultrasound platforms supports its potential for standardized use in perinatal pathology and forensic settings.

All fetuses in this study underwent feticide via intracardiac or umbilical vein injection of 2% lidocaine, which induces rapid cardiac arrest through sodium channel blockade [23]. This mechanism produces immediate anoxia with minimal ischemic delay, whereas spontaneous intrauterine deaths often result from progressive hypoxia due to placental insufficiency, cord accidents, or infection [24]. These differing agonal processes may affect tissue perfusion, dehydration, and autolysis, potentially leading to increased postmortem ocular volumes. Consequently, in cases of stillbirth, the PMI predicted by our model might represent a minimum estimate of the true death–US interval.

Interpretation of postmortem imaging must also consider the potential influence of pharmacologic agents before generalizing to spontaneous stillbirth. The two drugs most commonly used for feticide are potassium chloride (KCl) and lidocaine. Although KCl is not chemically corrosive, autopsy series have reported more pronounced tissue degeneration in fetuses receiving intracardiac KCl [25], and PM-MRI studies show accentuated soft-tissue changes compared with spontaneous stillbirths [26]. In contrast, systemic lidocaine is generally considered safe and effective [23,27]. Experimental studies have shown that direct intraocular injection can induce endothelial or stromal corneal changes, as reported in using 0.2 mL of 1% lidocaine hydrochloride [28,29]. However, systemic administration during feticide results in substantially lower ocular exposure. Therefore, while minimal ocular effects cannot be entirely excluded, the influence of lidocaine on corneal or ocular volume is likely negligible, supporting the applicability of our model to stillbirth.

### 4.3. Clinical Implications

Accurate PMI estimation allows precise determination of the fetal age at death, thereby supporting the evaluation of intrauterine growth restriction as a potential cause of demise [2,30]. PMI estimation also facilitates differentiation between true pathological findings and postmortem artifacts such as extreme umbilical cord narrowing or tissue dehydration.

In medico-legal and forensic contexts, objective PMI estimation provides valuable evidence for quality-of-care assessments and litigation when the timing of fetal death is uncertain [31]. Reliable PMI estimation enhances the diagnostic yield of postmortem investigations by correlating imaging, histologic, and placental findings [32] and supports clinicians in communicating clear and evidence-based explanations to bereaved families.

### 4.4. Research Implications

The model was trained using data from fetuses >20 weeks’ gestation. Therefore, PMI estimates for fetuses < 20 weeks remains imprecise. Future research should target the PMI estimation in younger gestations, evaluate model performance and its robustness in the presence of ocular malformations, and validate findings in multicenter cohorts.

### 4.5. Strengths and Limitations

This study has several strengths. The GBM model captured non-linear relationships among biological variables—including gestational age, birthweight, and the cornea-to-eyeball ratio—beyond what conventional linear regression could achieve. Training and validation followed standard machine learning principles, including fivefold cross-validation and independent prospective testing, minimizing overfitting. SHAP-based variable importance analysis ensured transparency, and consistent performance across two ultrasound systems supports reproducibility.

However, several limitations should be acknowledged. The study population included a heterogenous group of TOP indications, although only one fetus had open eyelids that prevented 3D PM-US acquisition. Application of the model in fetuses with severe ocular malformations or incomplete eyelids closure requires further investigations. The maximum death–US interval in our dataset was 147 h (approximately six days), reflecting the clinical workflow for termination and delivery induction. Therefore, the model is best suited for PMI estimation within the first postmortem week and should not be extrapolated to longer intervals. Extending the interval beyond one week was not ethically acceptable, as PMI values were derived from TOP cases to ensure precise timing of death. Consequently, a different predictive model would be required for PMI > 7 days.

Another limitation is that the study was conducted in a tertiary referral fetal medicine center with highly experienced operators and extensive experience in fetal virtual autopsy. Implementation of such technic in a new center would need previous training. However, the learning curve is expected to be short, as only fetal eye volume acquisition is required, and previous studies suggest that fewer than 30 cases are sufficient to achieve competence in full-body fetal PM-US examination. When PM-US is available, we recommend performing the examination within 72 h of delivery whenever feasible.

Finally, the single-center design, single-operator dependency and modest sample size may limit generalizability. Future multicenter and muti-operator studies, including centers with varying levels of PM-US, are needed to confirm clinical reproducibility and generalizability across different gestational ages and storage conditions.

## 5. Conclusions

This study demonstrates that 3D PM-US measurements of the fetal cornea and eyeball can reliably estimate the PMI with high predictive accuracy using a GBM model. Further multicenter studies are needed to refine the model, provide external validation, and further establish its clinical and forensic utility.

## Figures and Tables

**Table 1:** Baseline characteristics of the study population.

Variable	Training Cohort (*n* = 63)	Validation Cohort (*n* = 28)	Total (*n* = 91)
Gestational age, weeks	28.9 (26.5–32.0)	30.4 (27.8–33.3)	29.3 (27.2–32.9)
Birthweight, grams	1240 (925–1835)	1525 (1120–2192.5)	1325 (980–1880)
Male sex, *n* (%)	31 (49.2)	12 (42.9)	43 (47.3)
Death–US interval, hours	72.5 (50.8–114.2)	70.5 (52.1–114.78)	71.2 (51.0–115.1)
Delivery–US interval, hours	36.6 (21.4–78.6)	36.5 (14.7–69.0)	36.6 (19.6–78.6)
Eyeball volume, cm^3^	0.78 (0.56–1.00)	0.82 (0.67–0.90)	0.80 (0.59–0.97)
Corneal volume, cm^3^	0.05 (0.04–0.07)	0.07 (0.05–0.10)	0.06 (0.04–0.08)
Cornea-to-eyeball ratio	0.06 (0.05–0.10)	0.08 (0.07–0.11)	0.07 (0.06–0.10)

Values are presented as median (IQR) or *n* (%). Abbreviation: US: ultrasound.

**Table 2:** Confirmed fetal abnormalities leading to termination of pregnancy.

Diagnosis	Training Cohort (*n* = 63)	Testing Cohort (*n* = 28)
**Central Nervous System (CNS)**		
Agenesis of corpus callosum	5	2
Neural tube defect	9	0
Brain hemorrhage/hydrocephaly	11	4
Other brains pathology (schizencephaly/septal agenesis)	2	1
Cerebellar pathology	0	2
**Genetic and Metabolic Disorders**		
Tuberous sclerosis	2	2
Chromosomal/molecular aberrations	15	10
Cystic fibrosis	1	0
**Multisystem/Structural Anomalies**		
Polymalformation	9	0
Skeletal dysplasia	6	5
**Other Organ Pathologies**		
Congenital diaphragmatic hernia	1	2
Renal dysplasia	1	0
Fetal hydrops	1	0

## Data Availability

The data presented in this study are available on request from the corresponding author.
